# Business models for primary health care delivery in low- and middle-income countries: a scoping study of nine social entrepreneurs

**DOI:** 10.1186/s12913-021-06225-6

**Published:** 2021-03-09

**Authors:** Lutfi Lokman, Teresa Chahine

**Affiliations:** 1 Harvard T.H. Chan School of Public Health, Boston, USA; 2 Yale School of Management, New Haven, USA

**Keywords:** Innovation, Entrepreneurship, Social enterprise, LMICs, Primary health care, Delivery models, Universal access, Business models, Health information technology, Scaling

## Abstract

**Background:**

Social enterprises are organizations created to address social problems that use business models to sustain themselves financially. Social enterprises can help increase access to primary health care in low resource settings. Research on social enterprises in health care have focused either on high-income countries, or on secondary and tertiary care in low- and middle-income countries, where common business models include differential pricing to cross-subsidize low income populations. This is the first study to examine social enterprises providing primary health care in low- and middle-income countries using primary data. The purpose is to determine whether social enterprise is a viable model in this setting and to identify common patterns and characteristics that could inform the work of social entrepreneurs, funders, and researchers in this area.

**Methods:**

We identify social entrepreneurs working to deliver primary health care in low- and middle-income countries who have been vetted by international organizations dedicated to supporting social entrepreneurship. Through in-depth interviews, we collect information on medical processes, business processes, social impact, and organizational impact according to the Battacharyya et al. framework. We then conducted qualitative analysis to identify common patterns emerging within these four categories.

**Results:**

Common characteristics in the business models of primary health care social enterprises include flat rate rather than differential pricing and cross-subsidizing across services rather than patients. Subscription packages and in-house IT systems were utilized to generate revenue and increase reach through telemedicine, franchising, and mobile units. In some cases, alternate revenue streams are employed to help break even. About half of the social enterprises interviewed were for-profit, and about half non-profit. The majority faced challenges in engaging with the public sector. This is still a nascent field, with most organizations being under 10 years old.

**Conclusions:**

Social enterprise has been demonstrated as a feasible model for providing primary health care in low resource settings, with key characteristics differing from the previously commonly studied social enterprises in tertiary care. There are opportunities to complement existing public health systems, but most organizations face challenges in doing so. More research and attention is needed by researchers, governments and funders to support social entrepreneurs and avoid parallel systems.

## Background

Social entrepreneurship is an emerging field of research, teaching, and practice. Social entrepreneurship is defined as designing and implementing new products, services, and systems addressing social needs, to create a more just equilibrium rather than personal profit [1, 2]. Examples in health care have centered on organizations having a narrow clinical focus that may have facilitated experimentation with innovative delivery processes [3]. A commonly cited example is the Aravind eye care system, cited as the “McDonald’s of Health Care,” on which numerous research articles and teaching cases have been written [4]. Another similar example is Narayana Hrudayalaya Heart Hospital [5]. These specialized services are amenable to high volume, low cost business models, especially when the enterprise provides one primary product or service standardized across population segments. This allows for differential pricing to cross-subsidize low income populations, a common revenue model in social enterprise.

With the dire need for primary health care in low- and middle-income countries (LMICs), social entrepreneurs around the world are attempting to apply similar models to increase access to primary health care. Little is known of their business models and impact metrics; or whether similar business models to those used to-date in the delivery of more specialized clinical services, such as differential pricing, can be applied in primary health care with its wider range of services. LMICs typically have mixed health systems with the private sector accounting for more than half of health expenditures [6]. Higher income patients turn to for-profit providers as a more efficient alternative to government services [7], which often have long wait times and require lengthy travel, resulting in barriers to access [8, 9]. While the evidence indicates that this may not always be the case, for-profit providers were also often perceived to have better quality than government services [10]. When the public sector fails to provide quality primary health care for low income populations, they will either turn to for-profit clinics, clinics run by charities such as non-governmental organizations (NGOs) and faith-based organizations, or the newer and less common social enterprise clinics. Often, low income populations cannot afford visits to for-profit clinics, and hence are exposed to impoverishing health expenditures when urgency forces them to seek treatment there [11, 12]. Evidence also has shown that private for-profit clinics are less likely to operate in rural areas, even if their organization were supported by social franchising programs that target poor communities [13]. This is the gap that could possibly be filled in by social enterprises.

Social enterprises are organizations created to address social problems that use business models to sustain themselves financially [14]. In primary health care, social enterprise clinics differ from charitable clinics in that they find creative ways to earn profits to sustain themselves in the long term. Non-profit non-government providers, such as civil society organizations or clinics funded by church groups or national and international NGOs, gather no profit and depend on philanthropy to sustain their operations [15]. However, it is important to note that a social enterprise’s mission of providing care for underserved populations is as core to their success as any potential revenue, differentiating it from other for-profit clinics. A social enterprise clinic has an advantage in which they have full control of how they use their funds, whereas other non-profit clinics depend on grants and donations that are sometimes earmarked for specific purposes, such as the treatment of specific diseases that are prioritized by donors [16]. However, support for such clinics in the form of grants and donations is more widespread and recognized by the public. The lack of public exposure, recognition, and legal structure in lower- and middle-income countries makes finding venture support more difficult for social enterprises [17].

The literature on social enterprise in primary health care has focused on higher income settings such as the United Kingdom [18]. In England, community health centers were encouraged to ‘spin-out’ from public ownership into independently-run social enterprises with support from the government [19]. These demonstrated increased productivity, innovation, and responsiveness to underserved populations [20]. In Scotland, social enterprises improved health outcomes both through direct delivery of primary health care and community development programs addressing social vulnerabilities [21–23]. Often, in high-income countries, there exists specific legal entities for social enterprises to register, such as the Community Interest Company in the United Kingdom and the Social Purpose Company in Belgium. This encourages social enterprises to form networks and gain support the government [24]. In most LMICs, however, such legal forms do not yet exist, and hence social enterprises are often standalone institutions with little or no support from the government. There are only a few studies on primary health care social enterprises in LMICs and none have collected primary data [25, 26]. Battacharyya and colleagues investigated innovative healthcare delivery models in LMICs using secondary data and proposed a framework theorizing that these are characterized by interaction between business and medical processes driving organizational and social impact [3]. According to them, *business processes* are generic to most organizations, consisting of marketing, financing and operations; while *medical processes* are particular to health service organizations, including: prevention, diagnosis, treatment and monitoring. Interaction between these two processes produce two kinds of impact: *organizational impacts*, relating to the enterprise itself, including replicability and sustainability; and *social impacts*, relating to the beneficiaries, including availability, affordability, and quality of care. Tung and Bennett examined ten large scale private for-profit providers in LMICs and found that their business models had similar characteristics to non-profit providers, including social rather than commercial marketing, low-cost high-volume services, partnerships with government and differential pricing across customer segments. The majority provided specialized services such as eye care; and the authors found data paucity a challenge [25]. Angeli and Jaiswal examined six case studies in India using secondary data to identify business model innovations enabling inclusive health care. Results included co-creation of patient needs, community engagement, continuous involvement of customers, innovative medical technology, focus on human resources, strategic partnerships, economies of scale, and cross-subsidization [27]. The organizations included in this study provided secondary and tertiary care; hospitalization, emergency care, medical devices, or water and sanitation.

This study collected and analyzed primary data from a sample of social enterprises delivering primary health care in LMICs. This was motivated by the following practitioner-oriented research questions: can social enterprise be a viable model for providing primary health care in low resource settings? If yes, are there common patterns and characteristics of social enterprise in primary health care that could inform the work of social entrepreneurs, funders, and researchers in this area? To answer these questions, we conducted a scoping search using the portfolios of global organizations supporting social entrepreneurs, to identify those working in primary health care provision, and conduct interviews to gain insights into their operational and revenue models, financing, and impact metrics.

## Methods

This research was conducted at Harvard T.H. Chan School of Public Health from November 2017 through November 2018. We analyzed data from interviews with founders or executive officers of social enterprises delivering primary health care in LMICs according to World Bank classification [27]. Social entrepreneurs were identified from the following international organizations dedicated to supporting social entrepreneurship: Ashoka, Acumen Fund, Skoll Foundation, Duke SEAD, Echoing Green, Sankalp Forum, Schwab Foundation. While these organizations by no means encompass all social enterprises, they are well recognized for the breadth and diversity of the organizations they support worldwide and their accuracy and rigor in identifying and selecting them.

The social entrepreneurs were identified by reviewing the ‘health / health care’ portfolios. For the purpose of this study, we define primary health care as ‘the provision of integrated health care services that addresses a large majority of a patient’s personal health care needs - characterized by first contact, accessibility, longitudinality, and comprehensiveness [28].’ Based on this definition, we restricted our sample to those offering direct patient-provider relationship; excluding those focusing on enhancing the process of delivery (e.g. supply chain, technology or capacity building), financing access to care (e.g. community health insurance or vouchers), or transaction of health consumables (e.g. pharmacies or health stores). Founders or Directors/CEOs were contacted for an interview. We conducted semi-structured interviews using a topic guide based on the Business Models Innovations in Health Service Delivery framework by Bhattacharyya and colleagues [3] (Table 1). The objective of the interview was to 1) inquire what were the main processes involved in each organization with respect to their medical service and business operations. 2) discover the organizational and social impact that each of the organization has made 3) explore the relationship between the main processes – medical and business – to the social and organizational impact.

**Table 1:** Interview tool

Topic	Interview questions
Medical process	Can you describe the range of services provided by your organization? What are the most common services that patients are coming in for? How do you keep track of the health outcomes of your patients?
Business process	Can you describe the marketing strategies your organization employs? How does your organization maintain financial sustainability? How many funding sources you have today, and what is the main one? How did your organization first receive its initial funding?
Social impact	What does social impact mean to your organization? How does your organization measure social impact? How does your organization identify and assist low income patients? Do you collaborate with the public sector, if yes, how?
Organizational impact	How long has your organization been in operation? What were the key factors to your organization’s survival and growth? How far-reaching has your organization been in terms of people and geography? Has your business model been able to be replicated elsewhere?

With the exception of one in-person interview, all interviews were conducted by telephone. Interviews lasted between 25 to 45 min, with additional follow up by email. Interviews were conducted by the same researcher between June – August 2018. Audio recording was used to record the interviews. The same researcher conducted the interviews, transcribed the results, and organized into emerging themes. Coding results across the dataset was done using NVivo software. Emerging themes were then discussed and refined with the second researcher, resulting in the classifications presented in the results section below.

Validity and reproducibility of the results were ensured by: (1) Using a semi-structured interview topic guide (2) Recording the trail of data collection and analytic decision consistently using the software OneNote. (3) Confirming consistency of data from the primary transcript with online sources related to the organizations, including official websites and case studies. The multiple references from various resources provide triangulation in addition to increasing the robustness of our data. No repeat interviews were conducted.

This research design was submitted to the Office of Regulatory Affairs and Research Compliance at Harvard T.H. Chan School of Public Health and was determined as not Human Subjects Research due to the organizational rather than individual nature of the questions asked to interviewees.

## Results

### Sociodemographic and geographic characteristics of the social enterprises

Three hundred ninety-four social entrepreneurs were identified from the ‘health’ and ‘health care’ portfolios of the international organizations reviewed. This was narrowed down to 61 working in primary health care. The remaining 333 worked on specific diseases, tertiary care, and social determinants. Of the 61, 10 provide primary health care service delivery. The remaining 52 were excluded as they work in other areas of primary health care such as information technology, enhancing processes of primary health care, transportation to primary health care facility, funding primary health care, and direct-selling of health consumables. Of the 10 providing primary health care, nine responded to our interview request.

The nine social entrepreneurs were spread across four countries in three different regions: South Asia, Sub-Saharan Africa, and South America (Table 2). All the host countries of these social enterprises were classified as low- to -middle income countries by the World Bank. All the host countries share similar type of health service delivery that consists of a mix of public and private health facilities. The private sector consists of for-profit and non-profit organizations such as charities and social enterprises.

**Table 2:** Characteristics of sample organizations

Host country, region	Interviewee	Year founded	Business model	Initial funder
Kenya, Sub-Saharan Africa	Male, Co-Founder & Head of Product	2012	Low-cost, multiple chain of clinics, attracting high volume of patients. Focuses on maternal and child health.	Family & friends
Brazil, South America	Male, Public Relations Officer	2008	Multiple mobile medical centers. Low fees, attracting high volume of patients. Focuses on diagnostic procedures to shorten wait times in government facilities.	Family & friends
Argentina, South America	Male, CEO	1989	Low fees, multiple chain of clinics, attracting high volume of patients. No particular focus of primary health care service.	Family & friends
India, South Asia	Male, Founder & CEO	2014	Low fees, multiple chain of clinics, attracting high volume of patients. No particular focus of primary health care service.	Family & friends
India, South Asia	Male, Founder & CEO	2010	Low fees, multiple chain of clinics, attracting high volume of patients. No particular focus of primary health care service.	Family & friends
India, South Asia	Male, Founder & CEO	2012	Multiple mobile medical centers, low cost of monthly subscriptions. Focuses on chronic diseases.	Venture capital
India, South Asia	Male, Head of Quality Improvement	2008	Low-cost, multiple chain of regular clinics in peri-urban area and spoke clinics in rural areas. No particular focus of primary health care service.	Family & friends
India, South Asia	Female, Founder & CEO	2009	Multiple chains, annual subscription for chronic diseases where patient pays for the entire year of disease management. Low fees for acute diseases. Focuses on diabetes, hypertension, and hyperlipidemia.	Banks
India, South Asia	Female, Country Director	2008	Low-cost, multiple chain of regular clinics in peri-urban area and clinics with telemedicine in rural areas. No particular focus of primary health care service.	Family & friends

### Themes of the qualitative analysis

#### Medical process: curative versus preventive

Curative services were the main type of service offered in our sample organizations. The most common symptoms which these services were utilized for include fever, digestive symptoms, and respiratory symptoms, in which curative medications were mainly prescribed. Preventive services were carried out less frequently. The types of service offered by these organizations were largely based on demand from the patient population and were less guided by national policy frameworks of each country. For non-communicable diseases, two organizations reported doing health screenings for diabetes and hypertension as part of their main operation. Other organizations offered these services passively, to patients when there is a need, and to the community when dedicated funding was available. For communicable diseases, two organizations offer regular vaccination services, while others only offer vaccinations at the request of patients.

#### Business process: financial sustainability strategies

Strategies for financial sustainability included two components, cost reduction and income generation. Three common strategies were identified in each component (Table 3).

**Table 3:** Strategies for reducing cost, generating income, and increasing volume

**Financial sustainability strategies**		**Mechanism**
Costs	Bulk purchase of generic medicines	Purchasing generic medicines directly from suppliers allow organizations to negotiate lower prices as well as eliminating the cost incurred by intermediaries
	Paramedical staff	Training paramedical staff to treat simple cases lowers the cost of salaries
	Laboratory service	Running laboratory tests in own centralized laboratory saves the transportation and service cost of referring to outside laboratories.
Income	Flat-rate pricing	Charging a low flat-rate price to all patients save the direct and opportunity cost of measuring each patient’s socio-economic status
	Alternative revenue streams	Having alternative streams of revenue to support health services lowers cost to patients by subsidizing the cost of treatment
	Subscription packages	Subscription packages allows financial pooling that lowers the cost to patients by distributing the cost of treatment among all subscribers
**Scaling strategies**		**Mechanism**
Scaling physically		Chains, franchises and mobile units increase volume of patients by widening geographical access
Scaling virtually		Telemedicine increases volume of patients by increasing access to hard-to-reach areas and saving the cost of transportation

##### Cost reduction

Eight of nine organizations operate in countries with national health systems that did not reimburse cost of treatment in private facilities. These organizations obtained revenue directly from patients through out-of-pocket payments. Methods to keeping costs low were cross-cutting and included:

i. Bulk purchase of generic pharmaceutical products directly from manufacturer – Organizations were able negotiate a lower price and cut costs incurred by intermediaries. The bargaining power of the organizations were related to the size of their networks, which ranged from 7 to 30 chains/franchises. Six organizations cited this practice as the most effective method of lowering costs. One founder mentioned that their country’s status as the world’s largest producer of generic drugs played a substantial role in their ability to keep cost low.
ii. Use of paramedical staff to treat patients – Two organizations utilized paramedical staff such as nurses or medical assistants to attend simple cases to save cost in salaries.
iii. In-house laboratory services – One organization created a centralized laboratory to house diagnostic equipment, saving substantial costs compared to referring these procedures to outside laboratories. This was also the organization with the largest network of chains, at 30, allowing them to benefit from economies of scale from the laboratory.

Interviewees emphasized that being low-cost does not mean being the cheapest in the area. A more important aspect is to offer the lowest cost possible for high quality care:

*“I don't think it [our price] is really low. We are not the cheapest, we don't claim to be the cheapest, and we don't want to be the cheapest.”*

Male, Head of Quality Improvement, India

*“I would not say that we are absolutely the cheapest… we try to position ourselves as affordable to the mass market [in this country] as possible.”*

Male, Co-Founder and Head of Product, Kenya

Interviewees associated the low-cost of service fee with high volume of patients to become financially sustainable. Scaling mechanisms to achieve high volume are described below under organizational impact.

##### Income generation

i. Flat-rate pricing – Seven of the nine organizations did not differentiate price based on socio-economic status; patients pay a flat-rate consultation fees in addition to the cost of drugs and diagnostics. Two organizations differed. One systematically assesses socio-economic status using questionnaires and provide highly subsidized or free treatment to high poverty populations. The other identifies a location below the national poverty line and provides highly subsidized or free treatment at that location, charging higher fees in other locations. One founder pointed to flat rate fees as being ‘non-discriminatory’ towards all:
  *“When people come to our clinic, there is nothing like poor or rich. Every patient is the same. We don't differentiate based on income or anything, so the payment is the same for everyone.”*
  Male, Founder and CEO, India

The founder cited that charging a low flat-rate price to all patients saves direct and opportunity cost of measuring each patient’s socio-economic status.

ii. Alternative revenue streams – While most organizations exclusively run healthcare services as their revenue source, three organizations subsidize the cost of health services using alternative revenue streams. Two sell their self-developed health information technology systems (HIT) to other organizations. A third sells eyeglasses as an added revenue stream.
iii. Subscription packages – Three of the nine organizations offered subscription packages for patients with diabetes and hypertension. These models were similar in that they were a form of pre-payment where patients pay a yearly flat rate, regardless of their income status, to receive treatment and monitoring services for diabetes or hypertension. One of the organizations only offer subscription packages to other organizations like schools and religious centers in order to only receive group subscriptions. This mechanism was cited as a form of risk mitigation to ensure an adequate pool of financial resources during each enrollment in order to financially sustain the subscription program. All three organizations also only started the subscription package after 2 to 3 years of operation because they wanted to analyze the cost of treatment for chronic patients in order to inform their pricing structure. All three did not include coverage for hospital referrals. One founder cited this as a basic form of capitation payment that can keep costs low by cost containment-mechanism. Subscription packages were viewed as a basic form of insurance that allows financial pooling which lowers the cost to patients by distributing the cost of treatment among all subscribers.

#### Social impact

##### Management versus outcome metrics

All nine social enterprises measure social impact, but with variable metrics and level of rigor. The metrics generally fell into two categories: management and outcome metrics. All organizations measured management metrics including number of new patients, returning patients, and patient satisfaction scores. Outcome metrics were less common; the only one organization that measured outcomes used metrics specific to chronic disease, such as the proportion of chronic disease patients that has their disease under control. A top-management staff described the difficulty in measuring outcomes for acute diseases as they were only possible when the patient returns because of ineffective treatment or a new complaint. Active measurement of social impact for community members; regardless of whether they have ever utilized service or not, was viewed as difficult. A top-level management staff cited an ‘inherent trust issue’ when attempting to measure health trends in new communities. Another founder mentioned:

*“Our key indicators were primarily processes, it is extremely hard to measure outcomes at the population level to say that you know, yes, you have the impact. In terms of utilization, processes, and output indicators, we had many; but outcomes were very, very hard. Even though I stayed in the company for 8 years, we only had early signs within chronic care management that we were having a meaningful impact, but I cannot say that we have a population level outcome for the poor.”*

Female, Founder and CEO, India

Eight of the nine organizations defined ‘low-income’ based on geography, identifying all patients within the same vicinity as falling into low socioeconomic status. Hence, the impact of their organization towards low income patients was taken as the number of footfalls in their clinics. Only one organization assessed socio-economic status using questionnaires and hence impact towards patients living in poverty was able to be measured.

##### Access and patient base

Improving access to quality care through availability and affordability was a cross-cutting strategy to reach low income populations. A common strategy adopted by six out of the nine organizations was establishing their clinics in low income neighborhoods. One founder explained:

*“We really locate in little towns which are aggregation of villages. We are not present in larger towns. Most people that we had access to were lower middle class or poor people, the richer people were either going to big cities, or did not even stay in the area. So, I think given our core objective was access, where we located our clinics was the deciding criteria of who was our primary audience”.*

Male, Founder and CEO, India

Of these six organizations, only one operated in rural areas, and five are situated in peri-urban settlements. The remaining organizations that do not operate clinics in low income areas reach the underserved through mobile units, telemedicine and spoke clinics attended by clinical staff from the city on certain days of the week. Out of the nine organizations, six organizations were the only primary health care providers in their geographic areas. The remaining three organizations have several other competitions including government facilities and other for-profit clinics. One interviewee mentioned that there is a preference to go to their clinic because the patients still have to make certain amount of payments in government facilities.

*“Sometimes they go to the government, but even there they have to pay with a little bit of money. So, we position ourselves to be as affordable as possible for the population, like, with only one US dollar, you are able to see a doctor.”*

Male, Co-Founder and Head of Product, Kenya

The patient base for all organizations included all layers of society. All interviewees were not concerned about ‘misuse’ of low-cost services by the middle- and high-income population. Attendance of middle- and high-income patients indicated the good quality of services provided.

*“We don't ensure that everyone who is coming is poor. That is not our mandate. Our mandate is health for all. Even I go to the clinic, my boss goes to the clinic.* ***That is the perception that we want to change, that cheap clinics cannot be of good quality.*** *We want everyone from all strata of society to come to our clinic”*

Male, Head of Quality Improvement, India

*“Most of them are poor people, but they don't need to be poor people. Sometimes they just don't have access to health.”*

Male, Founder and CEO, India

#### Organizational impact

##### Scaling strategies

All interviewees were organized as chains, franchises, or multiple mobile units. Five organizations were chains of clinics, two were franchises, and two were solely operating through multiple mobile clinics. Chains and franchises ranged from 7 to 30 clinics, while mobile units ranged from 110 to 116 per organization. Having multiple chains increased the bargaining power for bulk purchase of medicines and allowed for economies of scale from in-house laboratory facilities.

Another scaling mechanism was ‘spoke’ clinics acting as peripheral extensions to main clinics, operating only on certain weekdays. Three organizations used telemedicine to connect patients in rural areas to doctors in main clinics.

Scaling was indicated as important for financial sustainability (Table 3). A top-level management staff mentioned the importance of multiple chains:

*“If we only have 5 or 7 branches, we overall lose money as an organization.”*

Male, Founder and CEO, India

Of the five organizations operating as chains, four achieved financial break-even. Two achieved positive cash flow 4 months into operation. Of the two that were franchises, both achieved break-even; as did the two that operated through mobile units.

##### Leveraging health information technology (HIT)

Broad and consistent utilization of HIT has proven to increase health care quality and effectiveness, reducing costs, preventing medical errors, and expanding accessibility [29]. All nine organizations utilize HIT in their operations. The common use of HIT among all organizations is the digitalization of patient information systems. Five organizations mentioned that this is important as it improved the efficiency of managing patients and allows patients to access any of their facilities. This is considered important because all the organizations operate either as chains, franchises or mobile units and the transfer or referral of patients from one facility to another is common. Three organizations developed their own patient information system, while others subscribed to a vendor. Three organizations use HIT in the form of telemedicine to increase accessibility and volume. One director emphasizes the role of HIT in her organization:

*“We really want to use tech in innovative ways… not to keep technology as the focus point, but technology as an enabler*.”

Female, Founder and CEO, India

Out of the nine organizations, only one organization, located in Brazil, fed patient information into the national health information database.

##### Challenges in engaging with the public sector

Eight of the nine organizations interviewed cited limited engagement with the public sector. These were limited to regulatory transactions such as reporting notifiable communicable disease, licensing of clinical facilities and renewing practice certificates for staff. Outside of regulatory engagements, one founder described their engagement with the public sector as one-sided:

*“We are helping them instead of them helping us, for example, when a public service has a problem, we go there with the doctors, like a health care mission”.*

Male, CEO, Argentina

One interviewee worked very closely with the public sector, whereby patients’ fees were reimbursed by national insurance schemes, the same medical record system was shared, and monthly audits were conducted for accountability. The organization’s main operations during initial establishment was providing primary health care services directly to underserved patients; as public sector engagement grew, the focus shifted to identifying the greatest needs of the public health system and tailoring health services to fulfill them. Today, alongside providing primary health care services, a big part of their operation consisted of efforts in shortening wait times for diagnostic procedures with backlogs in public facilities. This was done by providing those diagnostic procedures themselves and referring patients back to the public sector for continued care. Since these procedures were reimbursed by the national health insurance scheme, this became an important component of their financial sustainability. This level of engagement was described by a top-level executive to take a long time and huge effort to build trust:

*“After a long time, we start to have trust from the mayor, the secretary (of health), the (public sector) doctors. They started saying – You know, this [name of organization] is very interesting, I think they can do more.”*

Male, Public Relations Officer, Brazil

Of the three other organizations, only one other organization’s host country has such programs where the patients’ fees were able to be reimbursed by national insurance schemes. However, the organization did not establish the relationship with the government, citing distrust towards the public system as well as perception of inefficiency:

*“The public health care system is broken with corruption, you have a lot of delays, it is not optimum.”*

Male, CEO, Argentina

The categories elaborated above are summarized in Table 4.

**Table 4:** Summary of main results

Medical Process:	Curative services more common than preventive
Business Process:	Financial sustainability strategies include cost reduction and revenue generation (see Table 3)
Social Impact:	Focus on management versus outcome metrics Focus on increasing access in low income locations
Organizational Impact:	Use of physical and virtual scaling strategies to increase volume for both impact and sustainability (see Table 3) Leveraging HIT especially patient information systems to increase access, efficiency, and reduct cost. Challenges engaging the public sector

## Discussion

This is the first study examining the social enterprise model in a primary health care context using primary data in LMICs. We build on previous studies examining social enterprises in specialized care using secondary data. While there are similarities between the two, our results indicate key differences. These will inform efforts by social entrepreneurs, researchers, and funders working to increase primary health care access in LMICs.

A key difference was pricing mechanisms. The majority of specialized care social enterprises utilize differential pricing to subsidize across population segments depending on patient income. This is made possible by the standardization of a narrow scope of services which can be offered across population segments. Our results indicate that this may not be a feasible model for social enterprises providing primary health care, where the range of services is broader. Cross-subsidization across services is more feasible, leveraging services such as optometry, diagnostics, and HIT sales to subsidize clinical consultations.

The subscription model utilized by some interviewees could be an example of a revenue model for primary health care. This model has been described in a recent study by Leung et al., who examined a sample of organizations from the Center for Health Market Innovation database. One example was MicroEnsure, a for-profit venture operating in Sub-Saharan Africa and Asia. MicroEnsure partnered with a health plan in Tanzania, covering primary health care and limited secondary care for chronic diseases, maternity, and neonatal care for a union of coffee growers for about $2 USD per month [30]. This echoes previous results from Uganda, where a non-profit hospital tested a micro-insurance scheme to provide a stable source of funding [31].

While pricing mechanisms and revenue streams distinguished the primary health care social enterprises, several common traits remained. One was cost reduction strategies such as bulk purchasing of pharmaceutical and medical supplies and use of paramedical staff. The latter is especially a commonly documented cost reduction strategy in social enterprise in other sectors, coinciding with a push within public health to shift away from a physician-only model to a care team approach [32].

Other common traits related to scaling, to ensure a high-volume low-cost work stream. Of these, HIT was key. HIT could be a key to both sustainability and scalability of low-cost health services: interviewees that developed their own HIT managed more patients efficiently; reached remote populations through telemedicine and generated additional revenue through software sales. Thus multiple barriers to access were addressed including provider availability, direct transportation costs, and indirect costs from transit time. Mobile units, franchises and chains were further enabled by the use of IT platforms to track patients.

In addition to examining similarities and differences between primary vs. specialized care social enterprises, a key contribution of this study is characterizing common attributes of primary health care social enterprises in LMICs. One notable characteristic was that the majority provided curative rather than preventative services; suggesting that their function may be akin to urgent care clinics in high-income countries. This was surprising given the dire need for public health prevention, especially among low income populations. It may be that there is a more immediate, pressing demand for curative services, especially when loss of productivity and the ability to work is at stake. Our results indicate that the focus on curative type of services were driven by the demand of patients. Among low-income populations, awareness and knowledge on preventative care are low, leading to low demand of preventive services as well as the willingness to pay for these services [33].

This situation calls for a stronger engagement with governments to complement these services, as it is often part of the national health policy framework. For example, in Cambodia, government contracting of primary health-care services with non-profit organizations was proven to increase the proportion of children fully immunized in rural areas [34]. When it comes to preventative services, non-profit clinics in low- and middle-income countries are more likely to provide these types of services due to widespread funding from international donor organizations, and increasingly national governments, specific to those type of services [35]. This is not restricted to communicable diseases. Non-communicable diseases (NCDs) such as diabetes and hypertension are increasingly contributing the greatest burden of disease in LMICs and are increasingly a focus of government screening programs and national health policy frameworks. While the organizations interviewed did not play a leading role in screening for NCDs, our results suggest that they are contributing to improving access, quality, equity of service delivery for management of these diseases.

Despite the potential for social enterprises to complement government and and other not for profit services, they are struggling to engage with the public sector. Differences in public health systems is likely the largest factor in determining public sector engagement. The social enterprise which was most engaged with the government was in Brazil, while many of the others were located in India, Argentina and Kenya. The latter were also younger organizations. Thus, another reason could be the survival-mode of the younger organizations and their need to focus on establishing sustainable, scalable operations.

The creation of parallel systems has been a critique of social entrepreneurship in general and this is especially pertinent in public health, where strengthening health systems is a global priority [36]. The parallel system of primary health care social enterprises in low- and middle-income countries is in contrast with those in high-income countries, where social enterprises were supported by the government in the form of defining services packages and managing the quality of care [17]. If social enterprises can complement rather than replace existing health system offerings similar to the landscape in high-income countries, this helps address many of the above concerns. It is important to note that another distinguishing factor in high income country settings is the presence of meso-tier organizations that can provide support to front line care providers e.g. through accreditation, quality support, infrastructure support. Thus is it not only the maturity of the social enterprises themselves over time which could play a role in fostering public sector engagement as indicated above, but also the maturity of the ecosystem supporting social enterprises and other non-state providers.

Out of the nine organizations, only one has a form of government contracting in the form of providing diagnostic services to shorten wait times at government facilities. Evidence in low- and middle-income countries has shown that government contracting for primary health care services can be very effective. For example, in Bangladesh, a study comparing non-profit clinics with government contracting of nutritional services to those without government contracting show a more significant decrease in malnutrition rates in areas where the clinic with government contract was available. Hence, contracting with government is one possible way of effectively mediating this parallel pathway.

Another opportunity for engagement between social entrepreneurs and public health systems is in data collection. A noticeable result from our interviews was the focus on management metrics rather than health outcome metrics in all organizations interviewed. Interviewees cited time and cost as a prohibitive factor. One way to overcome these barriers is by investing in electronic health records (EHRs). Given that our results indicate that HIT was a critical success factor for social enterprises on multiple fronts, we recommend this as an area of focus for funders of social enterprises, and an area of potential collaboration with governmental stakeholders. Data collection systems could also play a critical role in tracking and ensure quality, a topic that was not touched on in our interviews. As social enterprises focus on scaling the volume for increased impact and increased sustainability, more data is needed on how they are ensuring consistency in quality of services. Like other private facilities in low- and middle-income countries, social enterprises largely operate outside of national regulatory frameworks, hence there is a need for independent ways to assure the quality of services provided. Tracking health outcomes is one possible way for initial funders and government stakeholders to impose better accountability mechanisms to social enterprises to improve health outcome metrics.

Previous research indicates that partnerships with government or support from social insurance schemes is a success factor for private for-profit health providers at the bottom of the pyramid [25]. This mirrors success stories from other sectors, such as the education sector [37]. New ventures and interventions first focus on piloting and demonstrating proof of concept, and in later years are able to advocate and integrate with existing institutions for long term growth. Our hope is that, as these social enterprise mature, and with the support of funders and government stakeholders, they will be able to integrate with and strengthen existing public health systems.

While our study is critical in informing efforts of social entrepreneurs striving to provide primary health care in LMICs, it should be noted that we applied a convenience sample which is neither comprehensive nor representative. We examined social enterprises identified and vetted by international organizations supporting social entrepreneurs, as a starting point to determine whether social enterprise is a feasible model in primary health care, and whether we could identify any preliminary patterns or characteristics. Furthermore, our interviewees were limited to the owners or employees of social enterprises. Interviews with relevant staff in Ministries of Health could have enriched and give a balanced perspective to our study. Our results make the case that this is an area which merits further attention by many relevant stakeholders including implementers, funders, and researchers.

Future research can build on these findings by including smaller scale local social entrepreneurs. Example we came across outside of our search include Hospitals Beyond Boundaries in Cambodia, which utilizes differential pricing to serve a subpopulation of ethnic minorities with cross-subsidization of different income levels; and eHealthpoint in India, which bundles services such as clean drinking water, medicines, diagnostic tools and telemedicine. Clinica Africa provides primary health care in Uganda using a for-profit model supplemented with donor support for rural areas through affiliation with an NGO. Another area for future research is understanding the failures. Out of the ten organizations approached in this study, the one organization that did not respond was a social enterprise which shut down. Learnings from such cases are invaluable in understanding what has and has not worked in different settings. Among the nine organizations interviewed, only two of the chief executives were women. While our interview guide had not included targeted questions examining gender discrepancies, future research merits focused attention to better understand this factor. Finally, more in-depth examination of organizations’ quantitative data on all four levels of the conceptual framework is needed to develop an advanced understanding of their models.

## Conclusions

In summary, social enterprise has been demonstrated to be a viable model to provide primary health care services in low resource settings. The social enterprises interviewed have increased availability and accessibility to quality care in LMICs using targeted strategies to shape their services, sustainability, social and organizational impact. The young age of the majority of organizations suggests that the field of social entrepreneurship in primary health care is still in its infancy. In shaping future resources dedicating towards understanding and supporting this sector, it is critical to understand how social entrepreneurs can complement and strengthen existing efforts to improve population health.

## Data Availability

The datasets used and/or analyzed during the current study are available from the corresponding author on reasonable request.

## Abbreviations

HIT: Health information technology
LMICs: Low- and middle-income countries
NGO: Non-governmental organization
